# Lessons learned with the Cobra prosthesis in elderly patients with complex distal radius fractures—a retrospective follow-up study

**DOI:** 10.1007/s00402-021-04101-w

**Published:** 2021-08-02

**Authors:** Stefan Benedikt, Peter Kaiser, Gernot Schmidle, Tobias Kastenberger, Kerstin Stock, Rohit Arora

**Affiliations:** Department of Orthopaedics and Traumatology, University Hospital Innsbruck, Anichstraße 35, 6020 Innsbruck, Austria

**Keywords:** Fracture, Hemiarthroplasty, Wrist, Geriatric, Salvage, Osteoporosis

## Abstract

**Introduction:**

Recently, the Cobra prostheses were introduced in the treatment of distal radius fractures (DRF) of elderly patients. Fracture prostheses provide an alternative treatment option for complex fractures where conservative therapy seems not acceptable and osteosynthesis seems not possible. Data reporting the feasibility of the Cobra prosthesis are sparse. Therefore, this retrospective follow-up study investigated the clinical and radiological mid-term outcome of the Cobra implant in complex DRFs of elderly patients.

**Materials and methods:**

Thirteen patients (mean age 73.5 years, range 65–87 years) were retrospectively evaluated with at least a 1-year follow-up after surgery. Objective and subjective clinical parameters as well as the radiological outcome and complications were analyzed.

**Results:**

The mean follow-up period was 31.2 months. Seven cases required a cemented prosthesis. The mean relative range-of-motion compared to the healthy side was 72.3% and 51.8% for extension and flexion, respectively, and 87.9% and 85.7% for pronation and supination, respectively. The mean grip strength was 78.3% compared to the non-operated side. Eight patients were very satisfied, five patients were partly satisfied with the result. The DASH, PRWE, MHQ and Lyon-Scores averaged 39.1, 36.2, 64.9 and 63.3 points, respectively. The mean VAS-Score for pain was 1.1 at rest and 3.2 during activities. Perioperative complications included one dissection of the extensor pollicis longus tendon, one heterotopic ossification, one radiocarpal dislocation and two cases of an ulnar impaction syndrome due to implant subsidence.

**Conclusion:**

The prosthetic treatment of complex DRFs in elderly patients with the Cobra implant led to clinically and radiologically satisfactory mid-term results. The Cobra prosthesis still does not represent a gold standard but can be regarded as a feasible salvage option for complex DRFs when osteosyntheses may not be possible and non-operative treatment will lead to further functional restrictions and wrist pain during performing activities of daily life in high functional demand patients.

**Supplementary Information:**

The online version contains supplementary material available at 10.1007/s00402-021-04101-w.

## Introduction

Distal radius fractures (DRF) are the second most common fractures among elderly patients over 65 years of age. They are mainly caused by a fall from standing height associated with a decreased bone mineral density [1]. Open Reduction and Internal Fixation (ORIF) is a well-established treatment option for those patients. With the advent of volar locking-plate systems and fragment specific fixation, surgeons have improved options for stable fixation of comminuted intra-articular fractures in osteopenic bone [2–6]. However, loss of reduction as well as intra-articular screw penetration with very distal fractures can still occur leading to malunion, osteoarthritis and clinical failure [7, 8]. On the other hand, low demand elderly patients often do well with nonoperative management of distal radius fractures even those with residual displacement [9, 10].

In severely comminuted cases of complex intra-articular fractures, surgical treatment with plate fixation and/or external fixation may not be possible and non-operative treatment may not be acceptable. In 2009, Roux [11] published first results of a wrist hemiarthroplasty with the SOPHIA implant (Biotech, Paris, France) as an alternative treatment option for complex DRFs with a severely destroyed radial joint surface in elderly patients. The more bone-sparing Cobra implant (Groupe Lépine, Lyon, France) was presented by Herzberg in 2015 [12]. Several further studies showed moderate short-term and mid-term results (3–127 months), so that primary hemiarthroplasty is currently seen as a salvage option rather than an accepted standard [11–20]. However, data reporting the clinical outcome are rare. Potentially some bias may emerge, because several reports mixed different prostheses designs in the clinical outcome measurement. Additionally, the inventors of the SOPHIA and Cobra prostheses, respectively, conducted all studies but one.

The aim of this retrospective follow-up study was an evaluation of the clinical and radiologic mid-term results of elderly patients treated with the Cobra prosthesis (Groupe Lépine, Lyon, France) for multi-fragmented intra-articular distal radius fractures with an irreparable joint surface.

## Patients and methods

Approval for conducting this study was obtained by the institutional review board. Fourteen patients (73.5 years, SD 6.5, minimum 65 years, maximum 87 years, 1 male, 12 females) with a DRF were treated using a hemiarthroplasty between April 2015 and March 2019 at our institution. One patient had already deceased because of a medical condition not associated to the trauma. The remaining 13 patients could be included into this retrospective analysis and were seen for a mid-term follow-up at least 1 year after surgery (on average 31 months, range 12–54 months).

**Fig. 1:**
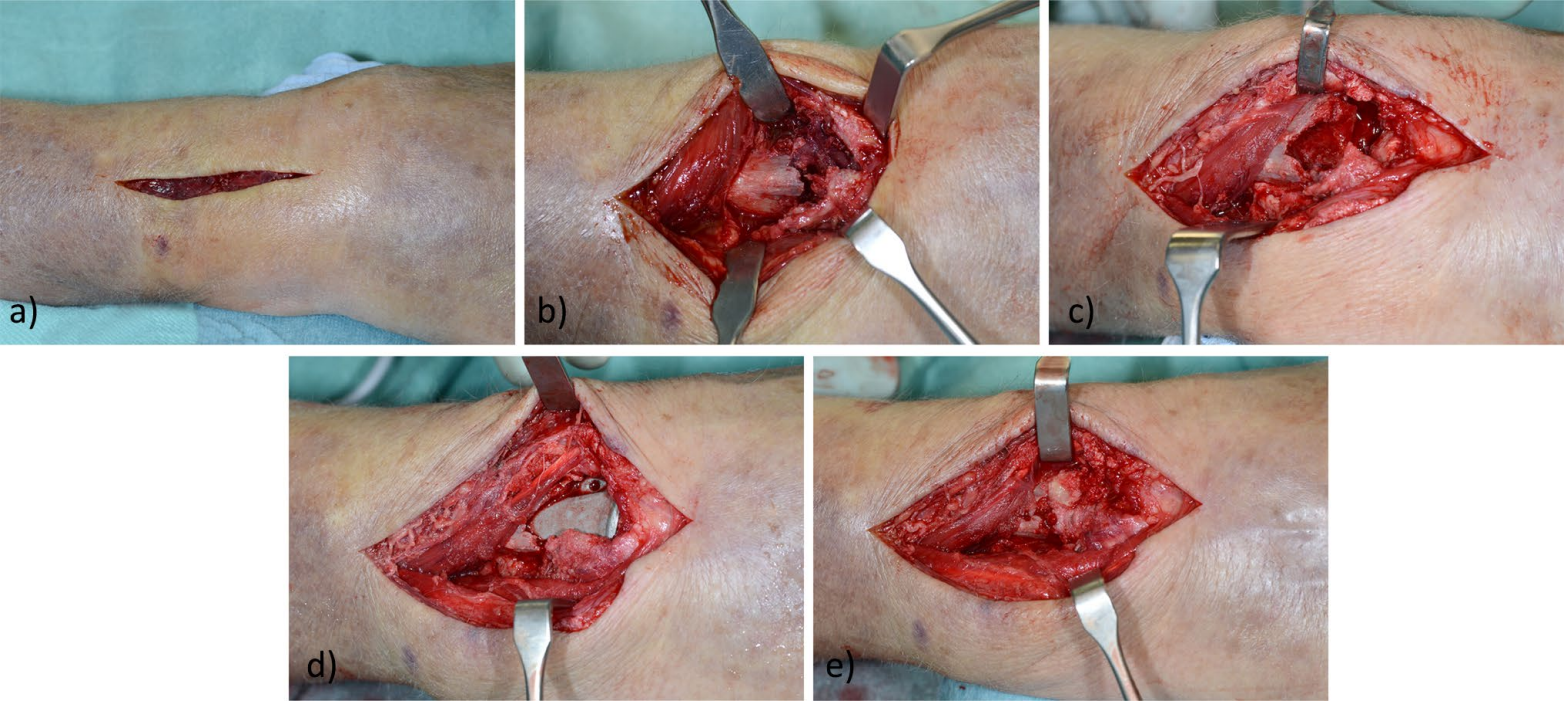
**a** Longitudinal dorsal approach in line with the third metacarpal bone. **b** Access to the comminuted distal radius by raising two osteoperisoteal flaps in an opening-book like fashion. **c** Distal radius after resection of the articular surface and the central epiphyseal cancellous bone and after broaching according to the preoperative planning. **d** Situation after implantation of the final prosthesis and reduction of the carpus. **e** The osteoperiosteal flaps brought back together

Indication for primary hemi-arthroplasty was set by an experienced hand surgeon in cases of unreconstructable comminuted AO-C3 fractures with distal fracture crossing the watershed line, separated palmar and dorsal fragments, impaction of the central joint surface and metaphyseal comminution. Additionally, the patients had to be aged 65 years or older and living at home performing activities of daily living independently. Surgical treatment was based on the technique described by Herzberg et al. [12, 21]. A longitudinal incision was made dorsally in line with the third metacarpal bone (Fig. 1a). The distal radius was accessed through the third extensor compartment. With an osteotome a longitudinal split of the radius was placed medial to the Lister tubercle up to the distal diaphysis and a radial and an ulnar osteoperiosteal flap were raised similar to opening a book (Fig. 1b). The radial part contained the second and the ulnar flap the fourth extensor compartment. A denervation was performed of the dorsal interosseus nerve. The articular surface of the distal radius and the central epiphyseal cancellous bone were resected. The volar, lateral and dorsal osteoperiosteal flaps of the distal radius were carefully preserved to save the bone stock and to allow bone covering of the prostheses. The wrist was then flexed to 90° and the diaphysis was broached according to the preoperative planning (Fig. 1c). The trial implant was gently inserted and a reduction of the carpus on the distal implant surface was performed. The radial and ulnar flangs were used to control the rotation of the implant. In cases of a too tight or too loose implant stability, reduction corrections by broaching or by inserting cancellous bone could be performed. In case of an appropriate reduction, the final implant was gently impacted into the distal radius (Fig. 1d). Bone cement was used if the implant stem could not be fixed stably at the metadiaphyseal junction. The osteoperiosteal flaps ware brought back together, sutured together (Fig. 1e) and the wound was closed.

Procedures to the distal ulnar were indicated as following: If the sigmoid notch was reconstructable, it was re-approximated and secured with non-adsorbable sutures and the distal ulna was left intact. A Sauvé-Kapandji or a Darrach procedure (depending on the surgeon’s preference) were performed in cases with severe involvement of the DRU-joint to prevent the ulnar head from erosions due to contact between implant and cartilage. For the Sauvé-Kapandji procedure a dorsal plate was used as the classical transfixation with a screw was not possible due to the position of the implant. The hemiresection-interposition technique described by Bowers [22] was not performed as it was considered not advantageous over the other procedures and authors did not want to take the risk of an impingement of the ulnar process and of a potentially associated revision in the already vulnerable patient population. In cases of open fractures, massive soft tissue swelling or other comorbidities that did not allow immediate hemiarthroplasty, a temporary external fixator was applied [23]. Postoperative treatment included cast immobilization and physiotherapy after cast removal.

Twelve cases had an irreparable DRF type AO-C3 and one case needed a secondary hemiarthroplasty after failed primary ORIF using a volar locking plate. The latter case was treated in an external hospital and was revised due to radiocarpal subluxation and consecutive joint destruction. Three patients had a grade I open DRF of whom one patient required primary treatment with an external fixator. The average time from injury to hemiarthroplasty was 9 days (SD ± 9 days) excluding the one case of secondary hemiarthroplasty 125 days after the trauma. Seven cases received a cemented prosthesis. In one case, a palmar approach was used, whereas in the other patients the recommended dorsal approach was used. Six patients received additional surgical treatment at the time of hemiarthroplasty: One patient had a re-fracture of the distal radius 9 years after ORIF with a palmar plate requiring implant removal and bone grafting beside hemiarthroplasty. The other additional procedures included the implantation of a dorsoulnar plate on the radius (*n* = 1); as well as a radial plate (*n* = 1); primary Sauvé-Kapandji procedures (*n* = 2) and a primary Darrach procedure (*n* = 1). The mean duration of surgery was 126.8 min (SD ± 55.2 min, minimum 63 min, maximum 250 min). Postoperative splint immobilization time ranged from 3 to 5 weeks; one patient had no immobilization. The average hospitalization time was 8 days (SD ± 5 days, minimum 2 days, maximum 21 days).

Detailed patient characteristics are listed in the Online Resource 1.

All patients underwent a clinical and a radiographic examination. Objective clinical parameters included the range of motion (ROM), the grip strength and the tip-to-palm distance. Patients were asked to reply to a German translation of the “Disabilities of the Arm, Shoulder and Hand Score” (DASH Score), the “Patient-Rated Wrist Evaluation Score” (PRWE Score), the “Michigan Hand Outcomes Questionnaire” (MHQ), the “Lyon Score” and to indicate their pain levels on the 10-point visual analogue scale (VAS) to evaluate the subjective clinical outcome. Overall satisfaction was rated by the patients having the choice between very satisfied, partly satisfied or not satisfied. Patients were also asked whether they would undergo the same surgery again, if necessary.

The radiographic examination consisted of a standard dorsopalmar and lateral radiograph at the follow-up. Radiologic outcome parameters included signs of periprosthetic osteolysis defined as radiolucency, implant dislocation, axial subsidence, osteoarthritis (OA) and erosions in the radiocarpal and the distal radioulnar (DRU) joint according to the Kellgren-Lawrence classification as well as measurements of the dorsal tilt, the radial inclination and the ulnar variance (UV) preoperatively.

Data are presented using descriptive statistics.

## Results

### Clinical results

Clinical outcome parameters are summarized in Table 1. Eight patients (62%) stated to be very satisfied and five patients (38%) were partly satisfied with the result. All patients would undergo the same operation again if necessary.

**Table 1:** Clinical mid-term results after hemiarthroplasty using the Cobra prosthesis

Measurement parameters	Mean (% of the uninjured wrist)	SD
Extension	46° (72%)	10°
Flexion	22° (52%)	13°
Radial deviation	17° (83%)	10°
Ulnar deviation	29° (71%)	11°
Pronation	67° (88%)	12°
Supination	69° (86%)	17°
Grip strength	17 kg (78%)	5 kg
Tip-to-palm distance	0 cm	0 cm
DASH	39	24
PRWE	36	23
Lyon	63	14
MHQ	65 (83%)	15
VAS resting	1.1	1.5
VAS working	3.2	2.2

*SD* standard deviation

The outcomes of patients having a Sauvé-Kapandji or Darrach procedure and of patients without intervention at the DRU joint are compared in Table 2. Patients with a Sauvé-Kapandji or Darrach procedure achieved better results in supination, grip strength and in pain during activities (also when excluding the cases with ulnar impaction syndrome). On the other hand, flexion, radial deviation and the DASH score were worse in this group.

**Table 2:** Comparison of the outcome of patients having Sauvé-Kapandji or Darrach procedure versus patients with no intervention at the DRU joint

Measurement parameters	Sauvé-Kapandji or Darrach *n* = 4		No ulnar intervention *n* = 9	
	Mean (% of the uninjured wrist)	SD	Mean (% of the uninjured wrist)	SD
Extension	50° (80%)	8°	44° (69%)	11°
Flexion	11° (31%)	16°	27° (59%)	10°
Radial deviation	9° (64%)	12°	21° (88%)	8°
Ulnar deviation	29° (79%)	13°	29° (68%)	11°
Pronation	61° (85%)	8°	69° (89%)	13°
Supination	78° (93%)	18°	66° (83%)	16°
Grip strength	15 kg (86%)	4 kg	18 kg (76%)	5 kg
Tip-to-palm distance	0 cm	0 cm	0 cm	0 cm
DASH	52	25	33	23
PRWE	39	30	35	20
Lyon	69	9	61	15
MHQ	62 (90%)	11	66 (80%)	16
VAS resting	1.0	2.0	1.1	1.4
VAS working	2.5	2.1	3.6	2.4

*SD* standard deviation

### Radiologic results

Preoperatively, the average dorsal tilt, radial inclination and ulnar variance were − 8.9° (SD ± 37.4°), 8.8° (SD ± 8.6°) and 6.1 mm (SD ± 4.1 mm), respectively. Postoperatively, signs of implant loosening were detected in two cases (around the stem *n* = 1; around the distal part of the implant shell *n* = 1). Radiocarpal signs of osteoarthritis were found in six cases but were not possible to quantify due to implant-related overlaps (sclerotic signs around the scaphoid *n* = 3; scaphoid erosions *n* = 1; radioscaphoidal synostosis *n* = 1; triquetral erosions *n* = 1). Among the nine cases without ulnar surgery, four cases presented sings of erosion due to the implant or signs of OA in the DRU joint (DRUJ erosions *n* = 2; OA grade I *n* = 1; OA grade II–III *n* = 1—according to the Kellgren-Lawrence Classification). Postoperative radiologic results of each patient are listed in Table 3.

**Table 3:** Postoperative radiologic results of each patient

Patient	Sex	Age (years)	Follow-up (months)	Cemented	Lucencies	Amount of subsidence (mm)	Signs of OA at the radiocarpal joint	Signs of OA at the distal radioulnar joint
1	f	66	12	No	No	0	n.a.^a^	No
2	f	75	12	Yes	No	0	Sclerotic signs scaphoid	Erosions
3	f	65	17	Yes	No	0	n.a.^a^	n.a.^b^
4	f	73	19	No	Around the shell	7.5	n.a.^a^	no
5	f	75	24	Yes	No	0	n.a.^a^	n.a.^b^
6	f	87	29	Yes	No	0	Sclerotic signs scaphoid	no
7	f	71	31	Yes	No	0	Radioscaphoidal synostosis	n.a.^b^
8	f	68	33	Yes	No	0	Sclerotic signs scaphoid	no
9	f	74	40	No	No	0	Triquetral erosions	Grade I^c^
10	f	66	42	No	Around the stem	0	n.a.^a^	n.a.^b^
11	f	76	54	No	No	0	Scaphoid erosions	Grade II–III^c^
12	f	80	54	No	No	4.5	n.a.^a^	Erosions
13	m	80	38	Yes	No	0	n.a.^a^	No

*OA* osteoarthritis; *n.a.* not applicable

^a^Due to implant-related overlaps in the X-ray

^b^After Kapandji or Darrach procedure

^c^According to the Kellgren-Lawrence Classification

### Complications

**Fig. 2:**
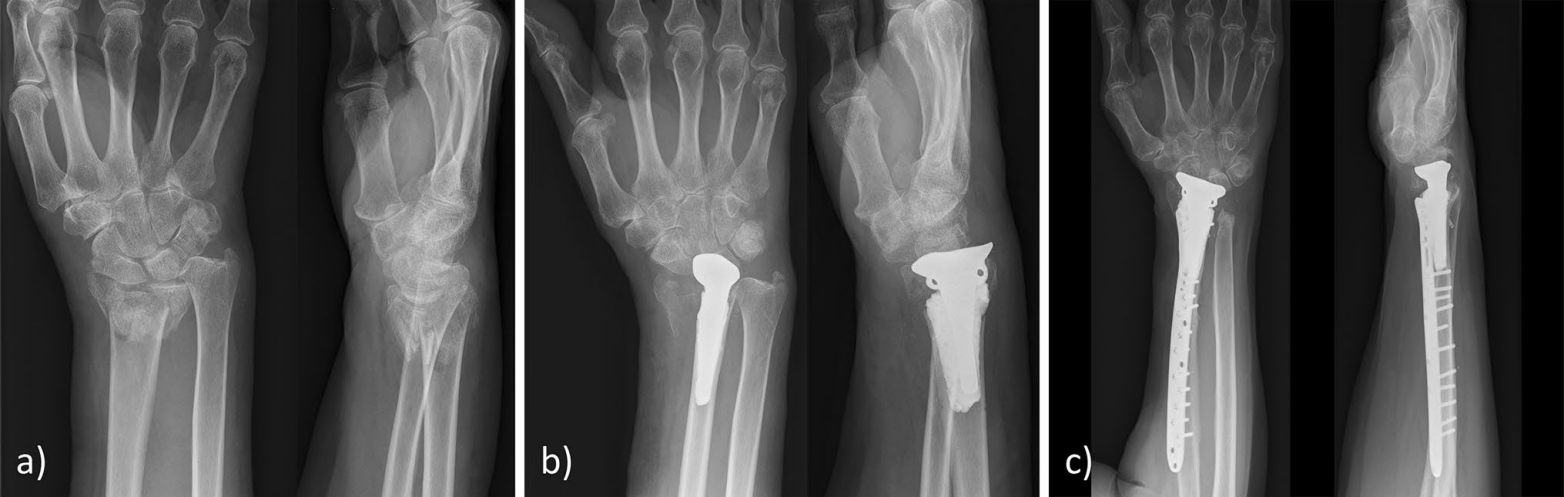
**a** Radiograph of the initial fracture. **b** Radiocarpal dislocation after hemiarthroplasty from a palmar approach. Revision included derotational osteotomy with a Darrach procedure and a pronator quadratus interposition transfer. **c** Radiograph at the follow-up 16 months after revision

**Fig. 3:**
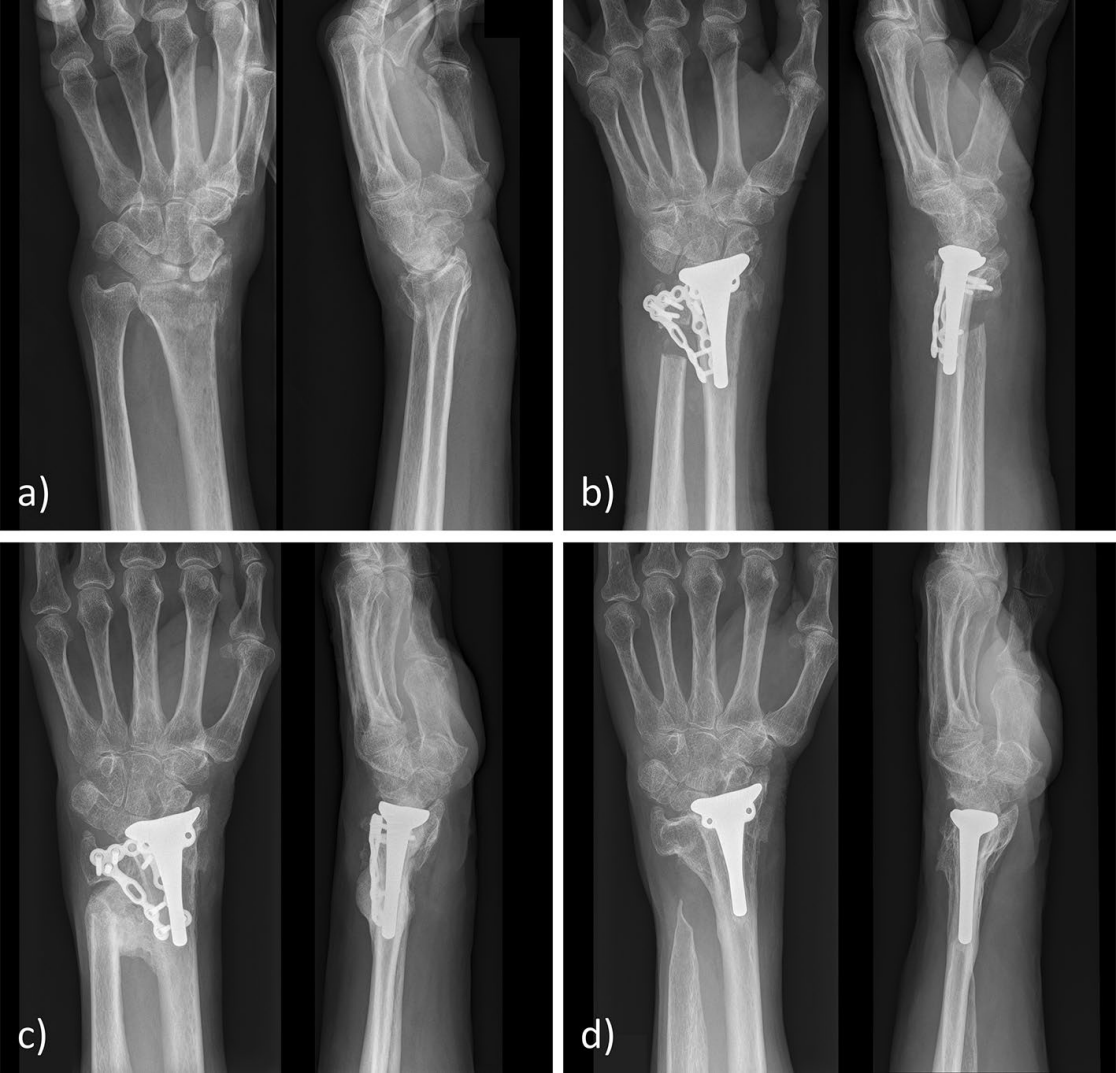
**a** Radiograph of the initial fracture. **b** Radiograph after hemiarthroplasty and Sauvé-Kapandji procedure with dorsal plating. **c** Severe heterotrophic ossifications 4 months after surgery leading to significant functional impairment. Revision included excision of the ossifications, removal of the dorsal radioulnar plate and a pronator quadratus interposition. **d** Radiograph at the follow-up 34 months after revision

**Fig. 4:**
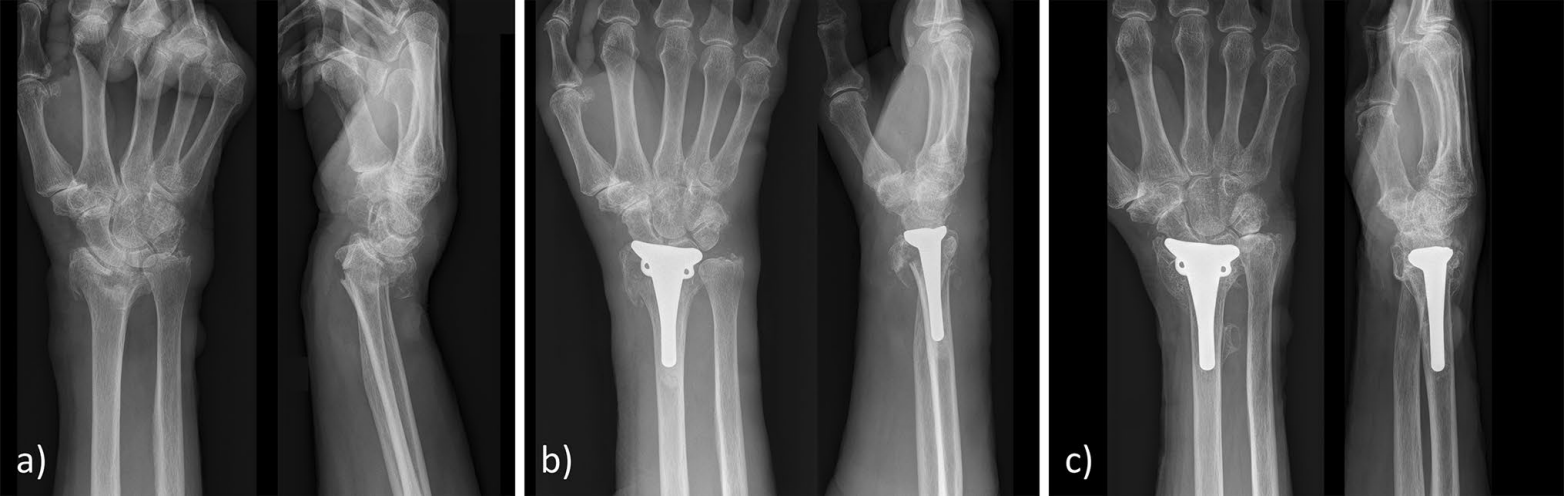
**a** Radiograph of the initial fracture. **b** Radiograph after hemiarthroplasty with a non-cemented prosthesis and successful reconstruction of the distal radioulnar joint with non-adsorbable sutures showing an acceptable position of the implant with a negative ulnar variance of − 1.5 cm. **c** Severe axial sinking with an ulnar variance of + 6.0 cm and clinical signs of an ulnar impaction syndrome 19 months after hemiarthroplasty

No patient developed a complex regional pain syndrome (CRPS) or an infection. An iatrogenic extensor pollicis longus (EPL) tendon laceration at the time of surgery occurred in one case. Two patients needed a secondary revision following hemiarthroplasty: The first case was the patient with the palmar approach who showed radiocarpal dislocation of the prostheses postoperatively. Initially, this patient was planned for ORIF. The result of the open reduction was not acceptable and therefore a cemented hemiarthroplasty was performed from palmar. The first postoperative X-ray revealed radiocarpal dislocation requiring a secondary derotational osteotomy of the radius shaft with a Darrach procedure and a pronator quadratus muscle interposition transfer at the proximal ulna stump (Fig. 2). The other patient with a Kapandji procedure developed severe heterotopic ossifications between the ulnar head and the distal stump of the ulna causing severe restrictions in rotation. The patient was revised nine months after hemiarthroplasty by excision of the ossifications, removal of the dorsal radioulnar plate and a pronator quadratus interposition transfer. Postoperative prophylaxis was not initiated, nevertheless, recurrence of ossifications could not be observed (Fig. 3). Axial subsidence of the implant was observed in two cases of non-cemented prostheses to an extent of an average of 6 mm (Table 3). One patient had a postoperative ulnar variance (UV) of + 2.0 mm postoperatively and + 6.5 mm at the final follow-up, the second patient had a postoperative UV of − 1.5 mm and + 6.0 mm at the final follow-up (Fig. 4). Both patients developed an ulnar impaction syndrome but did not request further surgical treatment.

## Discussion

The current mid-term results suggest that the Cobra prosthesis is a feasible option for complex distal radius fractures in elderly patients. The most important finding was the high satisfaction rate among all cases, with eight patients being very satisfied and five patients being satisfied with the result. All patients would also undergo the same operation again if necessary.

Encouraging objective results were achieved regarding ROM and grip strength except in flexion, which was on average 50% of the uninjured wrist. An explanation for the flexion deficit might be dorsal scar formation caused by the surgical approach. The mean VAS score showed on average mild pain with 1.1 at rest and 3.2 during activities. However, other subjective evaluation scores revealed obvious restrictions in daily life.

Another important finding was the axial subsidence of the implant in two of six cases of non-cemented prostheses leading to an ulnar impaction syndrome. Routine use of bone cement may probably avoid radial shortening.

A hypothesis to explain the better outcome in grip strength and pain in the ulnar intervention group might be the physiological relative increase in ulnar variance during grip [24]. In combination with radial shortening caused by the fracture and the hemiarthroplasty, activities that involve repetitive grip and forearm rotation might cause pain. However, given the small sample size of the groups, it is difficult to draw a clear conclusion from this data. A disadvantage of the ulnar interventions is the longer surgery time. Under favorable conditions, a Cobra prosthesis can be implanted much faster. The shortest surgery time among our cases was 63 min.

Hemiarthroplasty performed from a palmar approach has proven to be disadvantageous, as the reference points, especially regarding the rotation, for exact positioning of the implant are difficult to identify. The orientation of the prosthesis must be parallel to the anterior aspect of the radial epiphysis in the transverse plane. This can be achieved by the recommended dorsal approach more easily [21].

Recent studies showed moderate results for wrist hemiarthroplasty in elderly patients following complex DRF [11–20]. The idea of hemiarthroplasty for this indication was first implemented by Roux in 2005 [11]. The SOPHIA implant used in his patients is composed of a radial stem and an epiphyseal-metaphyseal block articulating with the carpus and the ulnar head. The clinical results of patients receiving hemiarthroplasty seem to be better and associated with a faster rehabilitation than reconstructive surgery (Table 4). The disadvantage of this design consists in a relatively large amount of bone material that has to be resected. Further, implantation is contraindicated in the event of a simultaneous fracture of the ulnar head or neck [11, 14, 17].

**Table 4:** Clinical outcomes of the SOPHIA implant

Study	Number of patients	Average age (years)	Follow-up time (months)	Pain (VAS)	Extension (°)	Flexion (°)	Ulnar duction (°)	Radial duction (°)	Pronation (°)	Supination (°)	Grip strength (% of the uninjured wrist)
Roux [11]	6^a^	73	27	1.5	65	30	20	20	60	50	80
Roux [14]	10^b^	75	29	n.s	60	36	26	21	67	61	72
Roux [17]	23^c^	77	56	n.s	62	37	26	31	72	68	79
Vergnenègre et al. [16]	8	80	25	2.3	44	45	25	20	85	75	92

*n.s.* not stated

^a^Five fractures and one malunion

^b^Six fractures, five malunions and one pathologic fracture

^c^17 fractures, five malunions and one pathologic fracture

Ichihara et al. [20] published a preliminary report of an Unicompartemental Isoelastic Resurfacing Prosthesis (Prosthelast, Argomedical, Cham, Switzerland) in comminuted articular DRF of osteoporotic elderly patients. The implant is designed as a joint surface replacement and is fixed with an intramedullary pin using an attachment screw. Advantages include the bone sparing properties and the intramedullary support that provides primary stabilization of the prosthesis [20]. A second report of the resurfacing prosthesis in the setting of distal radius fractures of elderly patients was published by Martins et al. [19] in 2020. Clinical results are summarized in Table 5.

**Table 5:** Clinical outcomes of the unicompartemental isoelastic resurfacing implant

Study	Number of patients	Average age (years)	Follow-up time (months)	Pain (VAS)	Quick-DASH Score	PRWE Score	Extension (% of the uninjured wrist)	Flexion (% of the uninjured wrist)	Pronation (% of the uninjured wrist)	Supination (% of the uninjured wrist)	Grip Strength (% of the uninjured wrist)
Ichihara et al. [20]	12	76	32	2.8	37	n.s	79	56	91	88	50^a^
Martins et al. [19]	24	78	55	2.1	40	43	73	55	97	88	66

*n.s.* not stated

^a^Neutral position

In 2015, Herzberg [12] reported first results of the Cobra prosthesis for complex DRFs, which is also more bone sparing and does not require an intact distal ulna compared to the SOPHIA implant. His experiences have been described in several publications [12, 13, 18]. A direct comparison to our current data is limited, as the authors combine the results of the Cobra implant with the radial component of the Press-fit-Remotion Total Wrist implant (Small Bone Innovations, Morrsiville, PA, USA) [12, 13, 18] (Table 6).

**Table 6:** Clinical outcomes of the Cobra implant

Study	Number of patients	Average age (years)	Follow-up time (months)	Pain (VAS)	Quick-DASH Score	PRWE Score	Lyon Score	Rotation (°) (% of the uninjured wrist)	Sagittal ROM (°) (% of the uninjured wrist)	Extension (°) (% of the uninjured wrist)	Grip strength (% of the uninjured wrist)
Herzberg et al. [12]^a^	11	76	27	1	32	25	73	151	60	34	67
Herzberg et al. [13]^a^	12	74	32	1	25	22	75	149	62	35	69
Herzberg et al. [18]^a^	27	77	32	1	26	25	74	150	60	36	68
Anger et al. [15]	11	80	18	3.8	59	72	50	164	63	27	44
Current study	13	74	31	1.1 at rest 3.2 during activities	39 (DASH-Score)	36	63	136 (87)	68 (64)	46 (72)	78

^a^Results combined with the radial component of the Press-fit-Remotion Total Wrist implant

In 2019, Anger et al. [15] published their experiences with the Cobra prosthesis in a retrospective study on eleven patients. The average age was 80.4 years, the average follow-up 18.3 months. Nine fractures were classified as C3, two were classified as C2 according to the AO classification. Eight patients received a cemented and three patients a non-cemented prosthesis. Two patients underwent an ulnar head resection at the time of hemiarthroplasty, one patient 6 months postoperatively due to rotational pain. Two patients developed a type 1 CRPS. The clinical results are listed in Table 6. Subjectively, seven patients stated to be satisfied or very satisfied, four patients were not satisfied. Radiologically, there was neither sign for an implant displacement or loosening, nor radial or ulnar tilt of the carpus.

Our current study had a longer follow-up time compared to the study of Anger et al. [15] (18 vs. 31 months). Subjective clinical results were notably better regarding satisfaction rate and the DASH, PRWE and Lyon Scores. Average pain was lower in our study, however, a direct comparison is difficult, as we assessed the VAS scores both at rest and during activities separately. Regarding the objective outcome we observed a better grip strength (78% vs. 44% of the healthy opposite side). An explanation for the difference might be the lower age among our study population. Sagittal ROM was nearly equal with a better extension but a worse flexion in our patients. Rotation was slightly better in the previous study. The number of cases with implant loosening, dislocation or axial migration was higher in our study. This might be explained by the higher number of non-cemented prostheses.

The results of the Cobra prostheses of the single studies are compared in Table 6.

An alternative to wrist hemiarthroplasty might be total wrist arthroplasty (TWA) which has been recently reported in two case reports [25]. It could be a viable treatment modality in cases of complex DRFs combined with high grade OA of the carpal bones, which is a contraindication for hemiarthroplasty [13]. Moreover, it would avoid the metal-on-cartilage contact between the implant and the proximal row. Given the numerous reports of cartilage damage due to increased shearing forces after hip-hemiarthroplasty [26], TWA might reduce the potential risk of erosions in the proximal row. One the other hand, TWA is a more invasive procedure and might therefore be more prone to complications. Radioscapholunate arthrodesis is a common modality in the treatment of osteoarthritis following distal radius fractures [27]. Arthrodesis as the primary therapy for DRFs was described sporadically around the year 2000 [28–30]. However, authors agree that function-preserving measures, with the shortest possible operation time, an as less invasive technique as possible and an early rehabilitation and reintegration into daily life should be the first choice in this population. Arthrodesis may still be a secondary option. Temporary internal fixation using spanning plates is another alternative for non-reconstructable DRFs [31]. However, in elderly patients this two-stage operation on one hand is prone to a higher perioperative risk (anesthesia risk, postoperative delirium, etc.) and on the other hand delays the functional rehabilitation, which is crucial for these patients.

Conservative treatment of distal radius fractures in elderly patients over 65 years has been subject of several recent studies [9, 10]. Most authors conclude that in an older population long-term clinical results are identical compared to surgical treatment. However, in the current cases of high-demand elderly patients with severe comminuted fractures, authors conclude that conservative treatment would have led to relevant restrictions in daily life.

**Fig. 5:**
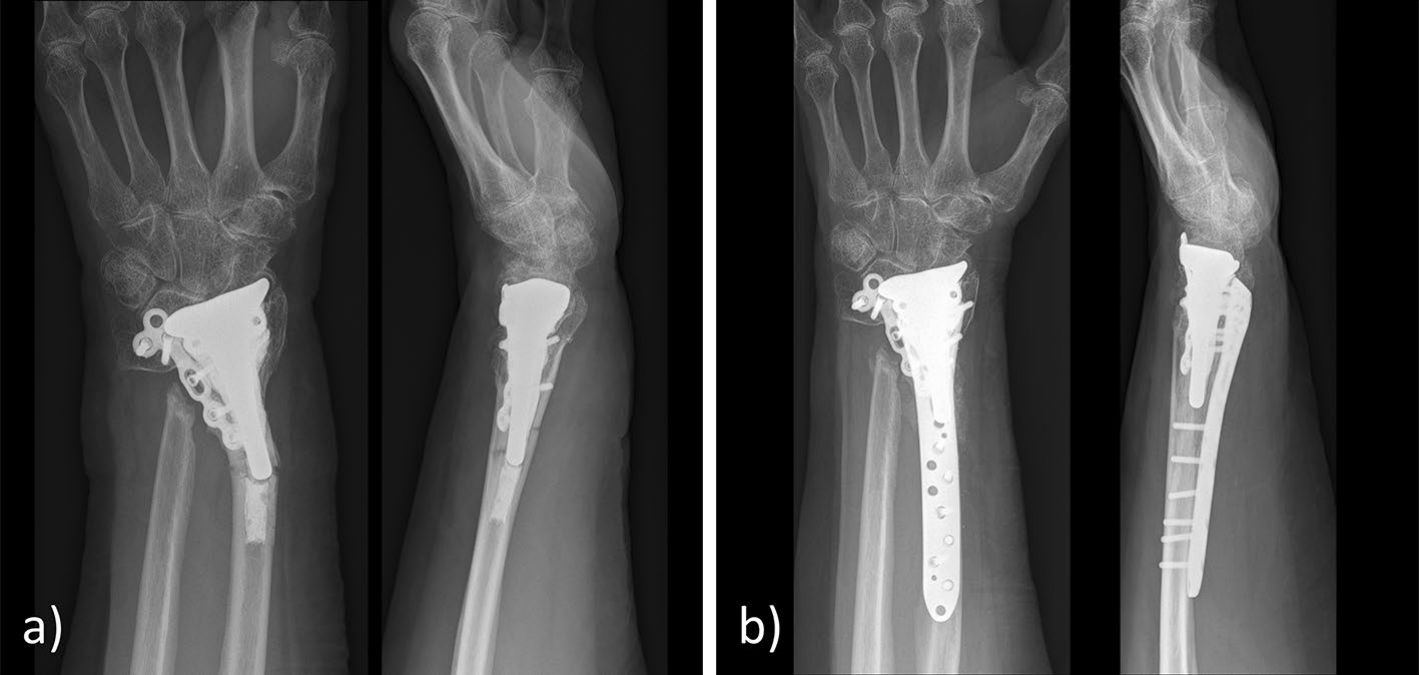
**a** Radiograph of a periprosthetic fracture after a falling accident. **b** Radiograph after revision by removing the cement from the proximal fragment, implantation of an allograft and osteosyntheses with a palmar plate

Finally, another important aspect of hemiarthroplasty in elderly is that the patients have a higher risk of falling. Complex periprosthetic fractures are therefore to be expected as shown in Fig. 5a. This patient (not included in the current study) was revised by removing the cement from the proximal fragment, implantation of an allograft and osteosyntheses with a palmar plate. The follow-up X-ray revealed bone union of the periprosthetic fracture without any signs of secondary dislocation (Fig. 5b).

Limitations of this study are the small sample size, the retrospective study design, the absence of a control group and the surgeon preferred treatment of the ulna head. Moreover, short-term follow-ups would have been useful to evaluate the early functional outcome and the re-entry into everyday activities.

Further clinical studies are needed to confirm our recent findings and to supplement the current data with long-term results.

The use of the Cobra prosthesis in complex distal radius fractures of elderly patients led to clinically and radiologically satisfying mid-term results. We learned that an essential point is the careful evaluation of the indications using bone cement and treatment of ulnar head. A palmar approach is not recommended due to missing landmarks. Personally, the authors prefer cementing the stem and in cases of an unreconstructable DRU-joint performing a Darrach procedure as it takes less time than the Kapandji procedure and can be realized without any additional implant. Anyhow, the surgeon must always keep in mind treating a frail population associated with a higher risk for complications and revisions related to poor bone quality.

The Cobra prosthesis still does not represent a gold standard but can be regarded as a feasible salvage option for complex distal radius fractures when osteosyntheses may not be possible and non-operative treatment will lead to further functional restrictions and wrist pain during performing activities of daily life in high functional demand patients.

## Supplementary Information

Below is the link to the electronic supplementary material.

Online Resource 1 Patient characteristics of the study population (PDF 259 KB)
