# Supplementary material for: Lessons learned with the Cobra prosthesis in elderly patients with complex distal radius fractures—a retrospective follow-up study
Source: Arch Orthop Trauma Surg. 2021 Aug 2;142(2):343–53. doi: 10.1007/s00402-021-04101-w (PMC8783885; doi:10.1007/s00402-021-04101-w)
Supplement: Online Resource 1 — Patient characteristics of the study population (PDF 259 KB) [file 402_2021_4101_MOESM1_ESM.pdf]

Online Resource 1: Patient characteristics of the study population

Patient	Sex	Age (years)	Time injury-prosthesis (days)	Follow up (months)	Injured side	Dominant side	Cause of injury	AO-Classification	Cemented	Time of surgery (minutes)	Anaesthesia	Hospitalisation (days)	Additional surgery at the time of hemiarthroplasty	Complications	Revisions	Multiple comorbidities[a)]	Comments
1	f	66	125	12	left	right	traffic accident	B3	no	250	GA	6	Implant removal, radial plate			No	Condition after primary palmar plating, secondary hemiarthroplasty due to radiocarpal instability and consecutive joint destruction
2	f	75	2	12	left	right	fall	C3	yes	140	GA	7	external fixator			No	open DRF
3	f	65	10	17	right	left	fall	C3	yes	120	GA	7		radiocarpal dislocation	Darrach procedure, derotational osteotomy, PQ-interposition transfer	No	
4	f	73	5	19	right	right	fall	C3	no	72	RA	9 (+rehab)		ulnar impaction syndrome		Yes	
5	f	75	3	24	left	right	fall	C3	yes	106	RA	10 (+rehab)	Darrach procedure			Yes	open DRF
6	f	87	1	29	left	right	fall	C3	yes	133	GA	8	EPL suture	EPL lesion		Yes	open DRF
7	f	71	7	31	left	right	fall	C3	yes	203	RA	5	implant removal, bone grafting, Kapandji procedure			Yes	Refracture nine years after initial palmar plating
8	f	68	9	33	left	left	fall	C3	yes	78	GA	2				No	
9	f	74	14	40	left	right	fall	C3	no	63	RA	3				No	
10	f	66	9	42	left	none	fall	C3	no	144	RA	16	Kapandji procedure	heterotrophic ossifications	HO excision, plate removal, PQ-interposition transfer	No	
11	f	76	35	54	left	left	fall	C3	no	93	GA	4				No	
12	f	80	14	54	right	right	fall	C3	no	140	RA	8		ulnar impaction syndrome		No	
13	m	80	3	38	right	right	traffic accident	C3	yes	99	RA	21 (+rehab)	dorsoulnar plate			Yes	

m…male, f…female, DRF… distal radius fracture, EPL… extensor pollicis longus, GA… general anesthesia, RA… regional anesthesia, PQ… pronator quadratus, HO… heterotrophic ossifications, rehab… rehabilitation
a) multiple comorbidities was defined by two or more chronic underlying medical conditions.

Title: Lessons Learned with the Cobra Prosthesis in Elderly Patients with Complex Distal Radius Fractures – A Retrospective Follow-Up Study

Journal: Archives of Orthopaedic and Trauma Surgery

Authors: Benedikt S, Kaiser P, Schmidle G, Kastenberger T, Stock K, Arora R

Affiliation: University Hospital Innsbruck, Department of Orthopaedics and Traumatology, Anichstraße 35, 6020 Innsbruck, Austria

E-mail of the corresponding author: rohit.arora@i-med.ac.at
